# Transport Distance of Invertebrate Environmental DNA in a Natural River

**DOI:** 10.1371/journal.pone.0088786

**Published:** 2014-02-11

**Authors:** Kristy Deiner, Florian Altermatt

**Affiliations:** Department of Aquatic Ecology, Eawag: Swiss Federal Institute of Aquatic Science and Technology, Dübendorf, Switzerland; Natural History Museum of Denmark, Denmark

## Abstract

Environmental DNA (eDNA) monitoring is a novel molecular technique to detect species in natural habitats. Many eDNA studies in aquatic systems have focused on lake or ponds, and/or on large vertebrate species, but applications to invertebrates in river systems are emerging. A challenge in applying eDNA monitoring in flowing waters is that a species' DNA can be transported downstream. Whether and how far eDNA can be detected due to downstream transport remains largely unknown. In this study we tested for downstream detection of eDNA for two invertebrate species, *Daphnia longispina* and *Unio tumidus*, which are lake dwelling species in our study area. The goal was to determine how far away from the source population in a lake their eDNA could be detected in an outflowing river. We sampled water from eleven river sites in regular intervals up to 12.3 km downstream of the lake, developed new eDNA probes for both species, and used a standard PCR and Sanger sequencing detection method to confirm presence of each species' eDNA in the river. We detected *D. longispina* at all locations and across two time points (July and October); whereas with *U. tumidus,* we observed a decreased detection rate and did not detect its eDNA after 9.1 km. We also observed a difference in detection for this species at different times of year. The observed movement of eDNA from the source amounting to nearly 10 km for these species indicates that the resolution of an eDNA sample can be large in river systems. Our results indicate that there may be species' specific transport distances for eDNA and demonstrate for the first time that invertebrate eDNA can persist over relatively large distances in a natural river system.

## Introduction

In order to understand community dynamics and biodiversity patterns, to protect rare species, or to mitigate consequences of range shifts, information on the current and past distribution of species is needed [1]. Thus, knowledge of where a species occurs is one of many fundamental variables of interest to the fields of ecology and conservation biology [2]. Many techniques to directly or indirectly detect species have been developed and applied [3]. These detection techniques range from visually observing the focal species (e.g., sightings of birds [4] or whales [5]), to collecting individuals through various kinds of trapping (e.g., emergence traps or kicknet-samplings [6]), or extrapolating presence from traces such as foot prints or feces [7]. Often, these detection techniques are very specific to the study organisms and cannot be applied across different taxonomic groups [2]. Additionally, many techniques depend on specific expertise that may be hard to learn or difficult to standardize, which can create unknown rates of false absences [8].

An ideal species detection technique [2] would be applicable to all species equally, would not depend on hard-to-define and hard-to-learn expert knowledge, would not depend on the removal of individuals from the population, and would have a way to systematically estimate false positive or false negative detections [9]. Environmental DNA (eDNA) is a novel molecular technique used to detect the presence of species and may have the potential to fulfill all of the above-mentioned criteria [10], [11]. Environmental DNA is a tracer method of detection and is carried out by extracting and identifying a species DNA from the air, water or soil in which it lives [10]. Thus, it is universal to all species (DNA), does not depend on the removal of individuals from the site, can be standardized to estimate false positive and negative detections, and can be carried out by trained technicians using standard molecular techniques. Currently, however, the calibration of this technique and establishment of the method is a rapidly developing field [10], [12]–[15] with many unexplored variables that affect detection of species from their DNA.

Use of eDNA methods for detecting species has been narrowly applied to some groups of organisms. In aquatic systems the taxonomic focus has been on larger vertebrate species such as fish and amphibians [12], [16]–[20] with a few studies extending this to invertebrates [13], [15]. Furthermore, the main hypothesis tested in many of these studies has simply been whether or not a species could be detected using their DNA found in water and tended to only speculate about the mechanisms for persistence, transport, and sources for the DNA. Therefore, many questions remain about the mechanisms that allow for a species to be detected with eDNA under natural conditions. Specifically, we need to address transport of eDNA, or cell/tissue fragments that can be the source of eDNA, in order to understand the spatial eDNA footprint of aquatic organisms in river systems. Such information is essential to promote and standardize this method of monitoring biodiversity in order to broadly apply it in flowing waters around the world.

**Figure 1:**
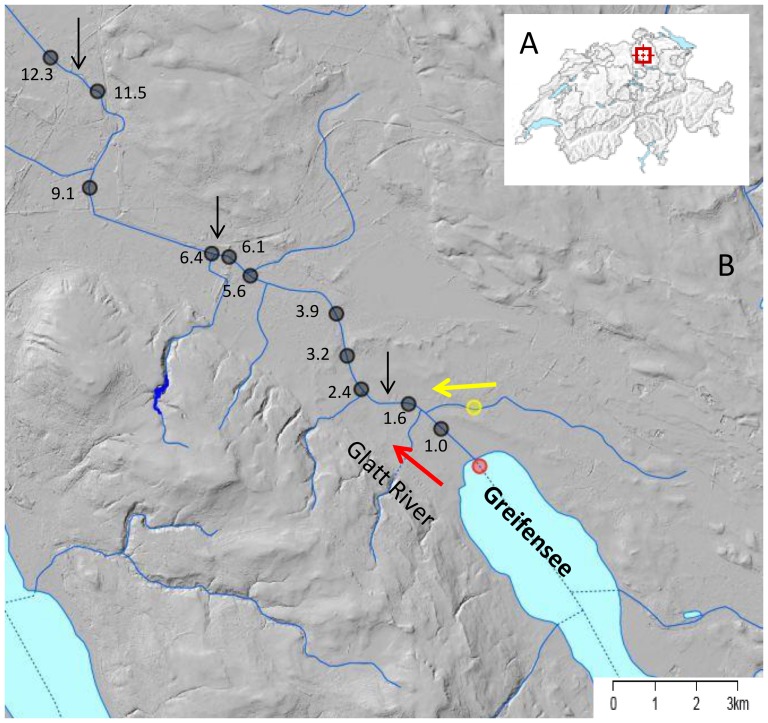
Depicted is the geographic area of study and river system where transport of eDNA was measured. Study area within Switzerland (A) and sampling locations (B) in the outflowing (direction indicated by red arrow) River Glatt. Red dot is the sampling location in the Lake Greifensee where the source populations for both species, *Daphnia longispina* and *Unio tumidus* are found. Chimlibach (yellow dot) is a small tributary feeding into the Glatt river system (direction indicated by yellow arrow) and served as a negative control. Black dots are sampling locations tested for presence of eDNA form the two species. Tributaries to the Glatt indicated in blue lines and additional dilution sources from wastewater treatment plant release points are indicated with black arrows. Numbers are the distance (in km) of the sampling sites away from the lake (measured as along-stream distance).

In this study we tested for downstream detection of eDNA for two invertebrate species, *Daphnia longispina* and *Unio tumidus*. *Daphnia longispina* is likely to be a species complex [21], but in this study we refer to the group here as *Daphnia longispina*. It is a planktonic crustacean (order Cladocera) and can reach up to 2 mm in length. It has a continuous, overlapping sequence of generations in summer, grows by regular molting, and can reach high population densities [22]. *Unio tumidus* is a sessile mussel (order Unionoida), inhabiting lake bottoms at relatively low densities and grows to about 10–15 cm in length. It is a long-lived, filter-feeding organism, producing planktonic gametes and larval stages (glochidia) only at specific times of the year [22]. Possible release of eDNA or tissue containing DNA (henceforth commonly referred to as “eDNA”) in these two species may occur during molting (where cells are shed off), production of mucous (especially for *U. tumidus*), production of small and easily dispersed reproductive stages, or decay of the organism when predated upon or after death. Both species are lake dwelling in our study area and have never been detected in the study river [23]. Our goal was to estimate the transport distance of eDNA for these two species by determining how far away from their source population in the lake their eDNA could be detected in its outflowing river (Fig. 1). We sampled water from eleven river sites in regular intervals up to 12.3 km downstream of the lake, developed new eDNA probes for both species, and used a standard PCR and Sanger sequencing detection method to confirm presence of each species' DNA in the river.

## Materials and Methods

### Study system and field collection

**Figure 2:**
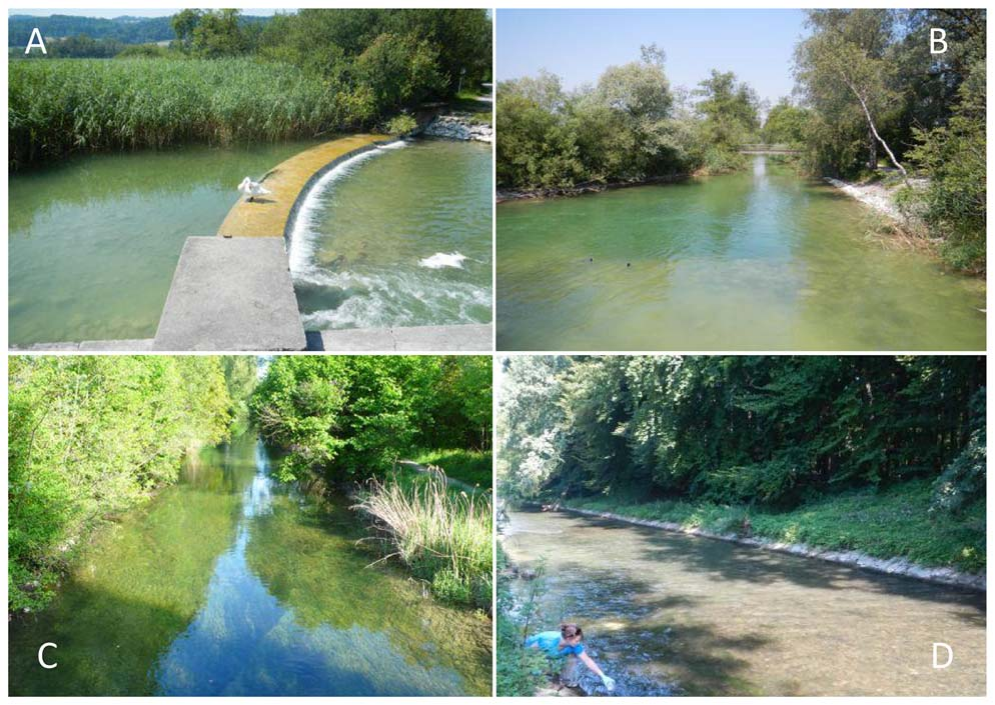
Illustration of river sites near or at collection points and demonstration of water sampling for eDNA. River Glatt at different distances downstream of Lake Greifensee outlet. A) lake outlet, B) about 0.5 km downstream, C) about 2.4 km downstream, and D) 6.4 km downstream, also pictured is K. Deiner taking the July water sample used in this study. All pictures except (A) are taken into the downstream direction.

Our study system was Lake Greifensee and its outflowing river Glatt (Fig. 1) in Switzerland. Greifensee is a eutrophic, pre-alpine lake with a surface area of 8.5 km^2^ and a maximum depth of 33 m. The outflowing river Glatt is human-modified and channelized (Fig. 2), with a 36 year (1977–2012) average water discharge of 3.79 m^3^/s in July and 3.52 m^3^/s in October [23]. The remaining riparian vegetation and the riverbanks of the Glatt are relatively homogeneous and it therefore offers an ideal setting to test the effect of river distance on detection rates of eDNA downstream of source populations because no other major environmental effects were expected within the study area (Fig. 1).

We sampled 900 mL of water by submerging a 1 L octagonal polyethylene terephthalate bottle (VWR International, Radnor, PA, USA) with a gloved hand just below the surface near the shore of 13 sites from downstream to upstream along the river (Fig. 1 and Fig. 2D). Bottles were purchased new for this study, had never come into contact with water from any sites before use, and were additionally pre-decontaminated before use by a 30 minute ultra-violet light treatment in a laminar flow hood in a DNA clean facility and sealed before use. Samples were stored on ice in the field, returned to the lab, the outsides of all bottles were decontaminated with 10% bleach, and stored in a −20°C freezer until DNA filtration and extraction was performed (maximum transport time was 4.5 hours). Black dots along the river represent the eleven sample locations used for the detection of eDNA where the species are not present based on long-term surveys that have been conducted by the cantonal nature conservancy agency [23]. In short, standardized samples for mussels have been taken with kicknets and were complemented by visual searching for mussels and waterfleas by experts at regular year to five-year intervals over the last 20 years [23]. Furthermore, there are no recreational activities, such as boating or rafting from the lake to the river, such that anthropogenic movement of these species is unlikely. In all of these surveys individuals of our two study species have never been recorded, which is also consistent with expert opinions on the ecology of these two species (personal communication by P. Spaak and H. Vicentini). Thus, the absence of these species has been solidified over a long time-period, and the species were also not found in the year where we conduced our study [23]. The lake sample site in red (Fig. 1) was considered the positive control for our study because both species have documented, well-established and long-lived (>20 years) populations in the lake [23]. The sample site in yellow (Fig. 1) is a small tributary that flows into the Glatt. It is not part of the drainage basin of Lake Greifensee, and was considered a negative control because no lake water flows into this stream. Furthermore, the two species have never been detected through traditional methods in this stream [23]. Sites were sampled at two time points, July 27, 2012 and October 29, 2012. Due to logistic reasons, not all sites could be sampled at both times. Sites 1.0 km, 1.6 km, 5.6 km and 9.1 km were sampled in both July and October. The positive lake control and sites 2.4 km, 3.2 km, 3.9 km, 6.1 km, and 6.4 km were sampled only in July. The negative tributary control and sites 11.5 km and 12.3 km were sampled only in October. All tributary streams to the Glatt along the sampling transect do not have a lake source, and thus could not be a potential source of the study organisms' DNA. We sampled before and after sites at which a dilution of eDNA may occur due to the influx of other water sources (e.g., natural tributaries or release point of treated wastewater, Fig. 1). We calculated minimal travelling time of particles in the water with the mean of the discharge values for the two time points reported above, an average river width of 14 m, and river depth of about 1 to 1.25 m.

### Probe design and optimization

Primer pair probes were designed using default parameters in Primer3 version 0.4.0 [24] from pre-existing sequence data available from the NCBI nucleotide database (Table 1, Dataset S1 and S2) [25]. Primer pairs were cross checked with alignment of available sequences, and when possible, placement of primer probes maximize base pair changes between the two closely related and co-occurring taxa in order to minimize amplification success of non-targeted species [20]. Primer sequences where then blasted against the NCBI nucleotide database using default parameters [25] for an *in silico* test of whether or not primers had a significant hit to the target species. Primer pair PCR annealing temperatures (Table 1) were optimized using extracted DNA from tissue of target species. PCRs on tissue extracted DNA were carried out in 20 µL volumes with final concentrations of 1x supplied buffer (Faststart TAQ, Roche, Inc., Basel, Switzerland) 1x BSA, 0.2 mMol dNTPs, 2.0 mMol MgCl2, 0.05 units per µL Taq DNA polymerase (Faststart TAQ, Roche, Inc., Basel, Switzerland), and 0.54 µMol of each forward and reverse primer. Tissue extracted DNA was added at 2 µL and ranged in concentration from 10–70 ng/µL. The thermal-cycling regime was 95°C for 4 minutes, followed by 35 cycles of 95°C for 30 seconds, either 50°C or 60°C (Table 1) for 30 seconds and 72°C for 1 minute. A final extension of 72°C for 7 minutes was carried out and the PCR was cooled to 10°C until removed and stored at −20°C until confirmation of products occurred. PCR products were confirmed by gel electrophoresis on a 1.4% agarose gel stained with GelRed (Biotium Inc., Hayward, CA USA). PCR products were cleaned using Exo I Nuclease (EXO I) and Shrimp Alkaline Phosphatase (SAP) (Thermo Fisher Scientific Inc., Waltham, MD USA). EXO I-SAP reactions were carried out in 8.5 µL volumes with a final concentration of 1.6 U/µL Exo I and 0.15 U/µL SAP. The thermal-cycling regime was 15 minutes at 37°C followed by 15 minutes at 80°C. PCR products were sequenced in both forward and reverse directions using dideoxy chain termination chemistry with Big Dye v3.1 following recommended ABI protocols and run on an ABI3730 automated capillary sequencer (Applied Biosystems, Foster City, CA USA). Forward and reverse sequences were aligned using Sequencher 4.9 (Gene Codes, Ann Arbor, MI USA). Consensus sequences were then aligned to sequences used for primer design (Table 1) to confirm amplified product matched that of the targeted region.

**Table 1:** Primer pair sequences and specifications used for detection of environmental DNA of targeted species.

Species	Primer name	Primer sequence 5′-3′	Gene	% divergent	Length	Mismatches in primer region	Product size**	Ta	Sequences used for primer design
Daphnia longispina	Dlong-F3	TGTATACCGCCGTTGTCAGA	12S	10	20	0	157	50	JX457151, EF375846, EF375851*
	Dlong-R1	ATCCACCTTCAACCAGCTTC			20	2			
Unio tumidus	Unio-F3	TACTGGTTGGACAGTATAC	COI	12	19	3	175	60	JQ253878, AF231732, JX046553*
	Unio-R2	AATCCGTTCAGCAACCAAAC			20	3			

* Mismatches in primer region come from comparisons with closely related co-occurring species used in primer design (*Unio crassus, Daphnia galeata*)

** Including target region and primers

### Environmental DNA capture, extraction, and detection

Water samples were removed from the freezer, defrosted and the outside of the bottles were treated with 10% bleach and transferred to a DNA clean facility that practices ancient DNA laboratory protocols in order to minimize potential contamination sources [26]. Water for each site was processed independently in a laminar flow hood. Each water sample was mixed by inversion five times, and an aliquot of 300 mL was poured into a beaker. The beaker was decontaminated with 10% bleach and subjected to 30 minutes of ultra-violet light before and after each water sample. Water was drawn up into a 20 mL disposable syringe and pushed through a housing containing a 0.22 µm glass fiber filter (25 mm diameter, Whatmen International Ltd., England). A total of 300 mL was passed through each filter and three filters were processed for each site to achieve a total volume of 900 mL. A negative control for the filtration process was created by using a filter that had been subjected to 30 minutes of ultra-violet light on both sides and filtering 300 mL of DNA-free water using the same filter housing and equipment as that used for all water samples and except that a new disposable syringe was used to draw up the DNA-free water. Each filter was then placed in a separate 1.5 mL microfuge tube and DNA was extracted using a modified cell lysis, phenol chloroform isoamyl procedure followed by an ethanol precipitation [27]. Briefly, 500 mL of a tissue lysis buffer (100 mM Tris-HCL pH 8.0, 5 mM EDTA, 0.2% SDS, 200 mM NaCl_2_) was added followed by 20 µL of Proteinase K (4 mg/mL) to each filter, mix gently by vortex for 10 seconds and incubated overnight at 55°C. Filters were removed and 450 µL of buffer equilibrated (pH of 8.0) phenol chloroform isoamyl alcohol (25∶24∶1, Sigma Aldrich Co., MO, USA) was added, samples were shaken manually for five minutes, centrifuged for five minutes at 10,000 rpm and the supernatant was pipetted off and transferred to a clean 1.5 mL microfuge tube. 450 µL chloroform isoamyl alcohol (24∶1, Sigma Aldrich Co., MO, USA) was added, samples shaken manually for five minutes, centrifuged for five minutes at 10,000 rpm and the supernatant was pipetted off and transferred to a clean 1.5 mL microfuge tube. 40 µL of 5M NaCl_2_ was added to each tube, followed by the addition of 900 µL of 100% molecular grade EtOH. Samples were placed at −30°C overnight to precipitate DNA. Samples were then centrifuged for 30 minutes at 10,000 rpm at 4°C, EtOH was poured off and 900 µL of 70% EtOH was added. Samples were centrifuged again for 30 minutes at 10,000 rpm at 4°C. EtOH was poured off, samples were air dried in a laminar flow hood for 15 minutes and DNA was re-suspended in 100 µL of AE buffer from the DNeasy Blood and Tissue Extraction kit (Qiagen GmbH, Germany). A negative control of the extraction was used to monitor any potential contamination during the extraction process. The three extractions from each site were pooled and stored at −20°C until PCR was carried out.

Detection of each species was carried out using the PCR protocol optimized for each primer set (Table 1) using the same protocol as above with the exception that 50 cycles instead of 35 were used to amplify the target. We tested three PCR replicates from each pooled extraction to determine a detection rate of the target DNA for each site. A PCR negative control was used for each PCR replicate. The negative filter, negative extraction, and negative PCR controls were used to monitor contamination at each step. For *U. tumidus* all positive detections in each replicate and for all sites were sequenced as described above (Dataset S1, replicates are labeled a, b, or c depending on which had an amplified product). For *D. longispina* we did not sequence all PCR products for each replicate due to the presence of a larger band in some replicates. We instead sequenced one replicate PCR product from each site for which no secondary band was present. This assured that for each site we had at least one sequenced amplicon, and guaranteed detection of this species at the site-level. Additionally, we tested whether or not the primers and PCR protocol used for *D. longispina* amplified the closely related and co-occurring species *D. galeata* (Dataset S2).

### Statistical analyses

We used generalised linear models (glms) to analyse detection rate of eDNA (i.e., proportion of positive PCR replicates per site) relative to three main factors: (1) the downstream distance of the sampling site from the lake (using along-stream distance), (2) species identity and (3) sampling time (July and October). We used a quasibinomial link function, as we had some overdispersion in the data, given the model, and an F-significance test [28]. The model initially included all main factors and their interactions. Residual deviance of models was used as the goodness-of fit criterion in the model-evaluation. The model was then hierarchically simplified, using F-test-based model comparisons in a stepwise algorithm, starting with removal of highest level-interactions first until we had one best model explaining detection rate of the eDNA by our three explanatory variables. All statistical analyses were done with the program R, version 3.0.1 [29].

### Ethics statement

No permits were required for the described study, which complied with all relevant regulations. Additionally, the subject in the photograph in Fig. 2 has given written informed consent, as outlined in the PLOS consent form, to publication of their photograph.

## Results

### Probe design and optimization

The *in silico* test for each species confirmed that primers blasted significantly to its targeted species. For *D. longispina*, the forward primer had no base pair mismatches, but the reverse had two base pair mismatches to that of the most closely and co-occurring species, and for *U. tumidus,* both forward and reverse primers had three base pair mismatches to that of the most closely and co-occurring species (Table 1; Dataset S1 and S2). Additionally, amplified and sequenced DNA from extracted tissues matched that of the targeted region for each species. No amplification was detected on the closely related species *D. galeata* when tested with the primers designed for *D. longispina*.

### Environmental DNA capture, extraction, and detection

**Figure 3:**
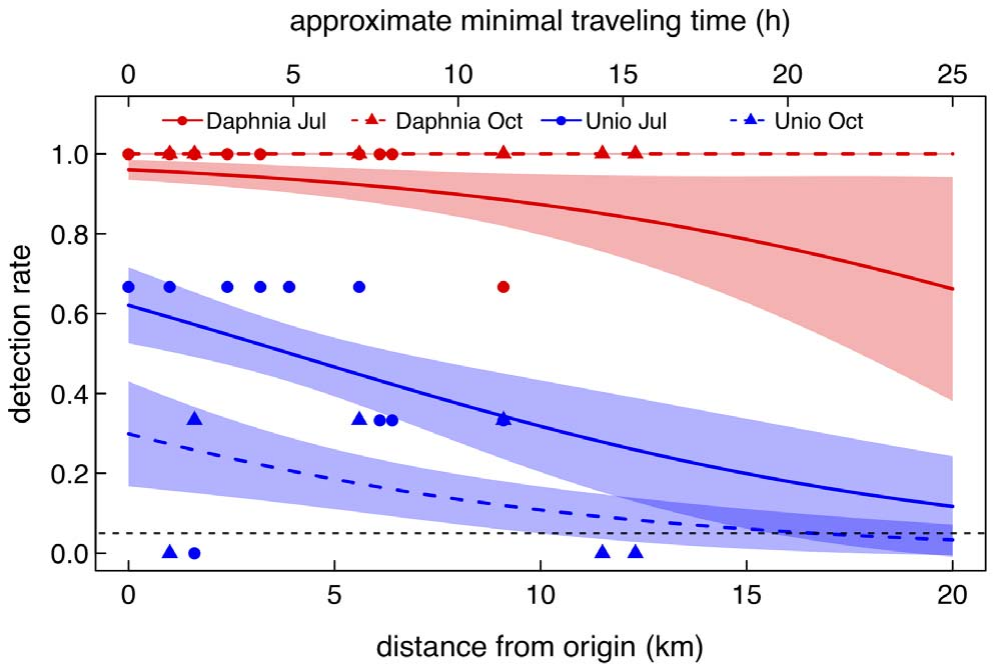
Observed and predicted detection of eDNA along river transect. Distance along the river Glatt from the source population (Lake Greifensee) at which eDNA for each species (red) *Daphnia longispina* and (blue) *Unio tumidus* was detected. Detection rate was determined as the number of positive amplifications of target DNA in three PCR replicates. The colored lines and the shaded area are glm model predictions (mean and standard error respectively) for the two species and time points (Jul: July and Oct: October) respectively. The black dashed line gives the 5% detection threshold. We also give calculated minimal traveling time of river water (and suspended eDNA and other particles therein) over the studied distances.

**Figure 4:**
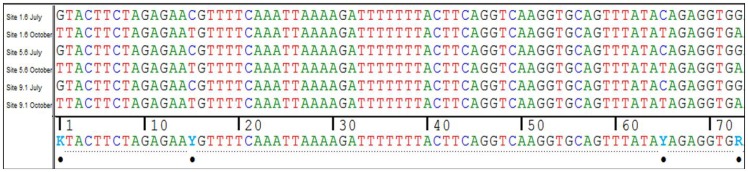
Detection of different haplotypes from sequenced eDNA. Sequence alignment of 12s amplicons from environmental DNA of *Daphnia longispina* showing different haplotypes detected between July and October at three sampling sites (1.6 km, 5.6 km and 9.1 km). Black dots indicated base changes between the two haplotypes.

*Daphnia longispina* was detected at all sites (except the expected negative control site) and across the two time points (Fig. 3). *Unio tumidus*, however, was not detected after 9.1 km downstream distance and was also not detected at 1.6 km in July, but was found in October at this site (Fig. 3). Also, and as expected, *U. tumidus* was not detected at the negative control site. While both species could be detected in the river, *D. longispina* had a positive detection for all three PCR replicates for all sites except 3.9 and 9.1 km away from the lake, where only two of the three replicates showed a positive detection. Sequences for the reverse direction of both species had a higher average quality value (81.4% *D. longispina*, 86.7% *U. tumidus*) to that of the forward direction (69.4% *D. longispina*, 79.3% *U. tumidus*). Additionally, for *D. longispina* the sequences from amplicons between the two time points indicated a shift in the haplotype detected from the environmental DNA that differed in four base pairs between July and October (Fig. 4). *Unio tumidus* on the other hand, did not have the same detection levels. Specifically, no site showed a positive detection in all three PCR replicates and there was a decrease in detection rate the further away from the lake the water sample was taken (Fig. 3). All filter, extraction and PCR controls were negative for targeted species eDNA.

We found significant effects of species identity, distance to source population and an interaction of time of the year and species on the detection rate (Fig. 3, Table 2). On average, detection rates were significantly higher for *Daphnia longispina* compared to *Unio tumidus*. For both species, there was an overall significant decrease in detectability with increasing distance (Table 2). Using the glm model to predict detection rates beyond our study distances, we found that the detection threshold of *Unio tumidus* falls below 5% at about 15 km and 25 km (fall and summer respectively) and for *Daphnia longispina* at about 50 km (summer). There was no decrease in detection rates over distance tested for *Daphnia longispina* in fall (Fig. 3), which precludes such predictions due to detection at all distances sampled.

**Table 2:** Generalized linear model, explaining detection rate of eDNA of two invertebrate species (*Daphnia longispina* and *Unio tumidus*) relative to along-stream distance from the source populations at two different time points of the year.

Estimate	Df	Deviance	Residual Df	Residual deviance	F-value	p-value
species identity	1	42.07	30	26.67	71.25	<0.001
distance	1	3.02	29	23.65	5.11	0.032
time point	1	1.42	28	22.23	2.40	0.133
species identity * time point	1	5.00	27	17.23	8.47	0.007
Null			31	68.74		

Df  =  Degrees of freedom.

Minimal traveling time of the river water was about 1.2 h for one kilometer. This gives traveling times of up to 16 hours to the most distant point sampled and does not consider the likely prolonged traveling time due to smaller flow rates at the bottom/shore line of rivers or due to turbulences.

## Discussion

Riverine systems are structured in a hierarchic network and this unique spatial structure influences flow of water, dispersal pathways of organisms, and biodiversity [30]. It may also be relevant to the distribution and use of eDNA as a monitoring tool in rivers. Specifically, in order to determine the geographic scale for a particular species using eDNA, it is essential to understand the transport distance of eDNA in a rivers' hierarchic network. Here we demonstrate for the first time that eDNA for two invertebrate species can be detected as far away as 9 to 12 km downstream from where their populations are known to occur. Our model predicts that the distance could be as far as 15 and 50 km before detection drops below a 5% threshold. Therefore, the geographic scale of an eDNA sample has the potential to be quite large. Several field studies of vertebrate species in lotic systems have related a local estimate of density using field detection methods to that of the detection probability estimated from eDNA for the species. They either found no relationship [16], [31] or a positive relationship [15], [17], [18]. Our results confirm a species' eDNA can be detected downstream from where it occurs and that the eDNA signal likely decays over distance. This is a possible explanation for why no correlation has been observed because drift of DNA from an upstream population would allow for downstream detection even when the downstream population is rare or not observed by a field observation method of detection.

Collectively, eDNA sampled from lotic systems indicates the presence of the species at local or upstream sites. The geographic scale, however, is likely to be on the order of catchments [18] and include both lentic and lotic species in the catchment if both water bodies are present. Therefore, by sampling eDNA at a single point in a river or stream, there exists the potential to retrieve an integrated measure of what is present in the contributing water bodies upstream. An integrated measure of biodiversity for catchments has the ability to transform monitoring data for whole systems. In most river and stream systems, characteristic biodiversity estimates come from point estimates retrieved from conventional monitoring methods, such as kicknet estimates of invertebrate biodiversity, and are extrapolated to represent the biodiversity present [32], [33]. The evidence from this study suggests that samples of eDNA could be used to estimate catchment biodiversity and that sample locations should be between 5 and10 km apart and follow the hierarchy of the network (e.g., sample nodes of confluence). A biodiversity estimate such as this would potentially reflect the dendritic network structure of rivers [30]. However, the exact distance between eDNA samples will vary by study system and is likely to depend on flow rates, size of the system, as well as the species specific rates of DNA shed into the environment, and the detection limit of primers used to detect an organisms' DNA. We calculated minimal traveling times of about 1.2 km per hour in our study system, allowing for a 16 h minimal traveling time of eDNA over the observed distances. This traveling time is based on transforming discharge and the river width/depth profile into an average velocity, and does not consider the likely slower velocities at the rivers bottom/bank, or delays due to turbulences. Effective traveling time of eDNA and retention of other cellular particles over the studied distances may thus be even larger, and in the range of 5 to 40 hours. Such time intervals may be long enough to affect DNA degradation and further decrease detection over a given distance [15].

The significant effect of species identity and species identity by sampling time interaction (Table 2) is a finding that may indicate some complications for the use of eDNA as monitoring technique across a wide range of taxa. Specifically, our findings suggest that either species' specific rates of DNA shed to the environment reflects either their different ecology or population dynamics, or that the specificity and ability of our primers to amplify the targeted species caused different detection rates among species. A species' specific effect is consistent with other studies, and the driving factors behind this effect remain unknown. For example, Thomsen et al. [15] found different detection rates of vertebrates and invertebrates in ponds, and Goldberg and colleagues [13] found that detection of amphibians varied by season similar to what we observed here. With our data, we cannot make a conclusive statement on the dependence of detection rates on species identity, especially for *D. longispina* since not all positive detections were sequenced. Primer specificity is not likely to be the main driving factor for the different detection rates because this would not explain the significant time by species interaction. We found that we could detect each species differently at different times of year and primer specificity is not expected to change over time. Rather the time by species interaction supports the claim that the way DNA is shed to the environment reflects different population dynamics/biology of the species and thus may affect detection by eDNA [16].

The significant difference in detection rates between the species could also be attributed to different population abundances for the two species, not having enough replicate PCRs, or (though unlikely) that false positives within sites were present for *D. longispina*. The number of three PCR replicates used in this study is similar to those from others (range of 3–8, [16], [34], [35]). We cannot post-hoc test whether the number of replicates impacts detection and recommend that future eDNA studies take this into consideration. The primers used for amplification of *D. longispina* did amplify a larger non-target product, and because of this, we were unable to confirm all positive PCR replicates through direct sequencing. However, the primers did not amplify the closely related species *D. galeata* when tested on the tissue extracted DNA and we did not observe the same non-target band amplifying consistently for each site. We therefore think it is unlikely that false positives were counted in the detection rate for *D. longispina*. Precise measures of population abundance are not available for our species in this study system, but we know from personal observations that the daphnid *D. longispina* is much more abundant in Lake Greifensee than the mussel *U. tumidus* (which is a rare species). We thus assume that part of the higher detection rates in *D. longispina* versus *U. tumidus* could be driven by abundance of the study organisms. An extension of this explanation is that *Daphnia* DNA may be dispersed more often through its predators via transport and defecation. However, the main unknown factor is how much and of what quality is the eDNA from individuals of the two species. We imagine that Cladocerans shed DNA into the environment by regularly molting, while mussels through the production of large amounts of mucus. Additionally, dead or dying *D. longispina* adults likely drift from the lake and are a potential source of eDNA that could exist for longer periods. Studying the species-specific (or life-history specific) shedding of DNA may be needed for standardizing detection rate comparisons among species, but also for making a more direct link between eDNA detection and population abundances.

In our study, we detected the planktonic species more often than the sessile, benthic organism. The mussel does have a planktonic gamete and a larval stage at some points of the year. We cannot exclude that part of the detected DNA was from whole organisms or gametes filtered from the water. We did not attempt to visually detect larval stages of *U. tumidus* on the filter, but we would have likely noticed large (>0.5 mm) *Daphnia* on the filters. Larval stages of either species are not likely to survive for very long in the river system, and for at least *Unio tumidus,* it does not produce gametes or larvae during the October-sampling period. Thus, detections for this species at this time point are most likely driven by the DNA derived from other cellular sources.

Most studies applying eDNA in lotic systems for non-microbe taxa have focused on large and mobile vertebrates species and whether or not they could be detected in flowing waters and mostly used a qPCR approach [15]-[18], [20], [36]. Very few studies have targeted invertebrate species [13], [15]. We encourage efforts to continue to expand the breadth of taxonomic groups and habitats explored with eDNA surveillance methods, such as done with this study, to demonstrate the universality of the method and establish it as an ideal method for describing species distributions. Additionally, while the use of qPCR for detection of species has its advantages, such as being a quantitative estimate of the targeted DNA from a total eDNA sample [20], use of standard PCR and sequencing methods can provide insights into population dynamics and genetic diversity within a species. We showed by sequencing the amplicon detected from eDNA that two different haplotypes of *D. longispina* could be detected at two different time points. *Daphnia* species are known to reproduce clonally and different clones can become more or less prevalent at different times of year [37]. Clonal changes in *Daphnia* species are attributed with having selective advantages and this may be driven by parasite loads [38]. Therefore, the detection of dissimilar clones at different times of year with the eDNA method highlights that it can potentially be used to monitor population dynamics and opens up a completely new application for eDNA.

## Supporting Information

Dataset S1 **Sequences obtained from eDNA for Unio tumidus.** This dataset gives the alignment of *Unio tumidus* sequences extracted from river water at different transport distances and time they were sampled. The a, b or c indicate the different PCR replicates from which the sequences were obtained.
(TXT)

Dataset S2 **Sequences obtained from eDNA for** ***Daphnia longispina.*** This dataset gives the alignment of *Daphnia longispina* sequences extracted from river water at different transport distances and time they were sampled.
(TXT)
